# Precocious markers of cardiovascular risk and vascular damage in apparently healthy women with previous gestational diabetes

**DOI:** 10.1186/1758-5996-6-63

**Published:** 2014-05-26

**Authors:** Lenita Zajdenverg, Melanie Rodacki, Janaina Polo Faria, Maria Lúcia Elias Pires, José Egídio Paulo Oliveira, Vera Lúcia Castro Halfoun

**Affiliations:** 1 Nutrology and Diabetes Section, Universidade Federal do Rio de Janeiro, Rio de Janeiro, Brazil

**Keywords:** Gestational diabetes, Cardiovascular risk, Microangiopathy

## Abstract

Previous gestational diabetes mellitus (pGDM) indicates future risk for type 2 diabetes (T2DM). Insulin resistance (IR) may precede T2DM in many years and is associated with an increased risk for cardiovascular diseases.

**Aim:**

This study aims to identify endothelial dysfunction and cardiovascular risk factors in women with pGDM.

**Methods:**

This cross-sectional analysis included 45 non diabetic women, 20 pGDM and 25 controls, at least one year after delivery. Body mass index (BMI), abdominal circumference (AC), blood pressure, serum lipids, liver enzymes, uric acid, nonesterified fatty acids, C-reactive protein and plasma glucose, insulin, fibrinogen and plasminogen activator inhibitor 1 were measured. HOMA IR and β were calculated. Pre and post induced ischemia videocapillaroscopy was performed in hand nailfold to evaluate microvascular morphologic aspect and functional response.

**Results:**

AC and fasting glucose were significantly higher in pGDM (p = 0.01 and p = 0.002 respectively). Women with pGDM and BMI < 25 kg/m^2^ had significantly higher levels of fasting insulin and HOMA IR than controls (p = 0.008 and 0.05 respectively). Abnormal morphologic findings were more frequent and papillae rectification were 3.3 times more prevalent in pGDM (p = 0.003). Other microvascular parameters did not differ between groups.

**Conclusion:**

Cardiovascular risk factors and a microcirculation abnormality (papillae rectification) were significantly increased in young non-diabetic women with pGDM.

## Introduction

Gestational diabetes mellitus (GDM) is a heterogeneous disorder defined as glucose intolerance first recognized during pregnancy [1]. This metabolic abnormality usually resolves immediately post partum, but implies in a higher risk of future type 2 diabetes mellitus (T2DM). In the vast majority of cases, the pathogenesis of GDM resembles that of T2DM. In both conditions, the β cell reserve is unable to counterbalance the insulin resistance. During pregnancy, this increased demand is caused by placental hormones. It has been suggested that GDM and T2DM should be taken as one single entity, although clinically evident in different lifetime periods [2].

T2DM is a known risk factor for cardiovascular disease (CVD) and insulin resistance (IR) has been implicated in this association [3]. The mechanisms involved in this link are multiple and complex. Chronic hyperglycemia may directly participate in the development of CVD [4] and other conditions frequently seen in patients with T2DM may also contribute to the increased risk, such as hypertension, dyslipidemia, altered fibrinolysis and obesity [5,6]. Endothelial dysfunction and chronic sub-clinical or low-grade inflammation are closely associated with obesity and insulin resistance and could be involved in the pathogenesis of T2DM and CVD. Increased levels of acute phase proteins such as C-reactive protein (CRP), interleukin 6 (IL6) and sialic acid have been shown in patients with T2DM and insulin resistance [7,8] and are known to be associated with CVD [8]–[11]. This low-grade chronic inflammation at the level of the vessel wall appears to mediate all stages of atherosclerosis and is a reflection of endothelial dysfunction. Capillary morphological and functional abnormalities have also been described in patients with T2DM during videocapillaroscopy (VC) [12-14], due to endothelial dysfunction, increased plasma viscosity, autonomic neuropathy and high vascular permeability [15,16].

Groups at risk for T2DM such as women with pGDM usually have more insulin resistance than the healthy general population, which can start many years before the appearance of any derangement in glucose homeostasis [17,18]. Beta cell dysfunction is also important for the development of T2DM in these individuals [18]. Ryan et al. have shown that women with previous GDM even with normal post-pregnancy oral glucose tolerance test (OGTT) had both insulin secretion and action defects in the post-conception period [19], although T2DM usually develops only years after pregnancy in this population. Therefore, this group might be considered in a very early stage of the natural history of T2DM and may offer a unique opportunity of studying abnormalities that appear early in the process.

It is still unclear how early a higher CVD risk appears in the natural history of T2DM. Women with previous GDM (pGDM) and a normal post-pregnancy OGTT already have a higher risk than the general population [20]. Endothelial dysfunction and sub-clinical inflammation have often been reported in individuals at risk for T2DM in general, which would contribute to the development of CVD [6]. In the specific subgroup of women with pGDM, few and contrasting data have reported vascular endothelial dysfunction and increased serum levels of inflammatory markers. Insulin resistance itself and increased abdominal fat could be implicated in these phenomena, although the possibility of a specific genetically determined vascular derangement has also been suggested [9,11,21-24]. The aim of this study was to identify cardiovascular risk factors and microvascular abnormalities diagnosed through videocapillaroscopy in apparently healthy women with pGDM from a multi-ethnic population.

## Patients and methods

We have performed a cross-sectional analysis of cardiovascular risk factors and endothelial function in women with pGDM and those with a past history of normal glucose tolerance during pregnancy. The protocol was approved by the Local Ethics Committee and informed consent was obtained from all subjects. Ninety six women who had the diagnosis of GDM between 24 and 30^th^ week of pregnancy at least 1 year before the study were invited to participate. The diagnosis of GDM was established with a 2 step- protocol according to the previous ADA criteria [1]. All patients were followed during and after pregnancy by the same medical team in the Diabetes and Pregnancy outpatient unit of Maternidade Escola da Universidade Federal do Rio de Janeiro. Women with T2DM, impaired fasting glucose or glucose intolerance according to OMS criteria [25], current pregnancy, menopause, use of vasoactive, antilipemic or antidiabetics drugs, vascular, kidney, liver, dermatological and infectious diseases were excluded from the analysis. From the 37 women with pGDM that accepted to participate, 20 were included. Twenty five healthy women selected in the same outpatient unit who had delivered 1 year or more before the study without pGDM, history of fetal macrosomia, menopause, use of vasoactive or antilipemic drugs and arterial hypertension were included as controls.

Maternal age in the labor, weight before pregnancy and in the third trimester, parity and previous abortions history were obtained in the medical charts; family history of diabetes, educational status, ethnic group, height, current weight, abdominal circumference and blood pressure were registered. Fasting capillary blood glucose (12–14 hours) was measured and subjects with results ≥ 140 mg/dl were excluded. All other patients underwent a 75 grams 2 hour oral glucose tolerance test. Serum creatinine, whole and HDL cholesterol, tryglicerides, liver enzymes, uric acid, nonesterified fatty acids, C-reactive protein and plasma insulin, fibrinogen and plasminogen activator inhibitor 1 were measured. Plasma insulin was measured with a fluoroimmunoassay (Auto Delfia kit, Turku, Finland). NEFA were measured with an enzymatic method (Wako, Richmond, VA); normal range in fasting state: 0.09-0.6 mmol/l). PAI-1 was measured with a Chromolize assay (Biopool International,Ventura, CA) wich reference interval for subjects between 20 to 49 years old was < 14.4 U/ml. Homeostasis model assessment (HOMA) IR and β were calculated. It was applied a logarithmic function of the HOMAβ in order to get a normal distribution of those values.

**Figure 1:**
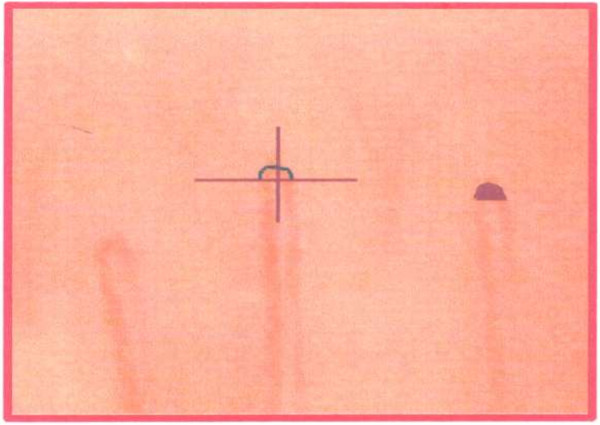
Determination of the capillary transverse segment area.

VC was performed in rest condition and during post-occlusive reactivity test and images were processed in a computer (Pentium 2 Microsoft). VC was done under controlled temperature environment (24–26°C) using microscope Wild Leitz GLS 100, videocamera COHU 8215, VCR and TV Sony 29-inch. Post-occlusive reactive hyperemia was performed using a sphygmomanometer attached to the fourth left hand finger, 20 mmHg above maximum arterial pressure during 1 min. Images were captured by a computer Pentium 1 (Microsoft) through Scanpro 3 software, each 2 s, during 1 min after releasing pressure. Measures were determined through Scanpro 3 software by at least two investigators blind to clinical data and in two different moments by each investigator to establish the concordance (*k* > 0.6 in both). A perpendicular line tangent to internal limit of a capillary loop transverse segment defined the transverse segment area (TSA) to be measured as shown in Figure 1. Zero time point after ischemia was considered just after releasing finger pressure. Occasionally, temporary minimum hand movement could not be prevented, blurring the image area and leading to higher surface values. In those circumstances, those measures were not included for the purposes of the work. Basal area of transverse segment of the capillary (ATSb), maximum area of the transverse segment of the capillary(ATSm) obtained ≥ 60 seconds after the occlusion, time to get the maximum post-ischemia area (MAIt) and the maximum increment percent (MAIp) from basal measurement were determined in patients with pGDM and controls. The following morphologic microvascular abnormalities were also investigated through the evaluation of the recorded basal images by two independent examiners: granular flow (clumps of red blood cells inside the capillary), disordered distribution of loops (when the disposition of the capillary loops was not positioned parallel to the edge of the nail, as a fence), loops of aberrant format (with the aspect of a hair clip with tortuous efferent and rectilinear afferent branches), short loops (when there was an area of edema partially erasing the branches), microectasy (saccular dilation in the capillary branches), papillae rectification (erasure or fusion of two or more papillae), tortuous transverse segment and enlarged transverse segment. The transverse segment unites the afferent and the efferent branches of the capillary loop. Tortuosity was defined as the presence of spiral movement and capillary distention.

For the statistical analysis, Mann Whitney U and Fisher exact tests were used to compare data between groups. Spearman correlation was used to test for association between continuous variables. All tests were two-tailed, with *α* = 0.05 for the main hypothesis.

## Results

### Clinical evaluation

The study group comprised 45 women, 20 with pGDM and 25 controls, who were evaluated 43.26 ± 29.17 months after the delivery, in average (range: 12–144). Their main characteristics are shown in Table 1. Groups were similar in age, number of previous pregnancies, ethnicity and number of school years. No differences were found among eight women in use of oral contraceptives (five in pGDM and three in controls), when studied separately. Two women in each group were smokers and all denied alcohol consume.

**Table 1:** Characteristics of the study group

***Variables***	***Previous GDM Mean (SD)***	***Controls Mean (SD)***	***P value***
Mean age (in years)	33.5 (4.9)	33.3 (6.7)	0.93
Ethnicity			0.37
Whites	8 (40%)	14 (56%)	
Non-whites	12 (60%)	11 (44%)	
Educational level (years)	9.4 (3.1)	10.5 (4.4)	0.50
Mean number of labors	2.0 (0.99)	2.1 (1.1)	0.96
Mean number of pregnancies	2.3 (1.22)	2.4 (1.4)	0.99
Age at conception (years)	31.3 (4.3)	29.1 (6.4)	0.17
Nursing period (months)	17.7 (15.6)	14.3 (12.1)	0.49
Body mass index	29.7 (5.9) Kg/m^2^	26.5 (6.19) Kg/m^2^	0.13
Abdominal circumference (AC)	102.3 (16.3) cm	91.2 (16.2) cm	**0.01**
Systolic blood pressure	114.5 (17.9) mmHg	110.8 (16.5) mmHg	0.60
Diastolic blood pressure	76.5 (11.8) mmHg	72.8 (12.0) mmHg	0.28
N	25	20	

SD = Standard deviation.

A family history of T2DM (in first degree relatives) did not differ between groups (45% vs 48%; p = 1.0). There were also no significant differences between groups in the blood pressure or the body mass index (BMI), as seen in Table 1. Two women with pGDM reported a previous history of hypertension and one used hydrochlorothyazide irregularly. In control group, only one woman had history of hypertension with no specific treatment. A higher abdominal circumference (AC) was observed in pGDM when compared to controls (102.3 ± 16.3 cm vs 91.2 ± 16.2 cm; p = 0.01) and AC > 88 cm was twice more common in the former than the latter. There was a correlation between AC values and weight gain after the index pregnancy (r = 0.53; confidence interval: 0.09-0.8; p = 0.018), but not with weight gain during pregnancy (r = -0.3; IC:-0.67-0.19; p = 0.21). Women with pGDM had a tendency towards lower HDL levels (46. 5 ± 11.4 vs 53.6 ± 11.0 mg/dl; p = 0.06) than others. Uric acid levels, transaminases, total and LDL cholesterol, triglycerides, free fatty acids (FFA)**,** CRP, fibrinogen and PAI-1 levels did not differ between groups. These results are shown in Table 2.

**Table 2:** Laboratory results

***Test***	***Previous GDM Mean (SD)***	***Controls Mean (SD)***	***P value***
Mean fasting glucose	83.2 (13.0) mg/dl	71.6 (9.4) mg/dl	**0.002**
Creatinine	0.72 (0.1) mg/dl	0.67 (0.1) mg/dl	0.14
AST	24.4 (5.2) U/l	24.9 (4.9) U/l	0.71
ALT	35.6 (7.8) U/l	40.8 (13.6) U/l	0.25
Uric acid	3.9 (0.7) mg/dl	3.7 (0.8) mg/dl	0.36
Total cholesterol	194.0 (33.8) mg/dl	185.6 (43.8) mg/dl	0.35
HDL c	46.5 (11.4) mg/dl	53.6 (11.0) mg/dl	0.06
LDL c	130.0 (32.3) mg/dl	114.3 (34.9) mg/dl	0.10
Triglycerides	87.0 (38.1) mg/dl	88.1 (63.1) mg/dl	0.44
FFA	0.47 (0.19) mmol/l	0.55 (0.23) mmol/l	0.21
CPR	0.34 (0.23) mg/dl	0.40 (0.49) mg/dl	0.50
Fibrinogen	279.5 (58.7) mg/dl	297.1 (84.7) mg/dl	0.50
PAI-1	12.1 (9.6) U/ml	16.0 (12.0) U/ml	0.25
N	25	20	

SD: Standard deviation; AST : Aspartate aminotransferase; ALT: alanine aminotransferase; HDL: High density lipoprotein; cLDL: Low density lipoprotein, c FFA: free fatty acids, CPR: C-reactive protein, PAI-1: plasminogen activator inhibitor-1.

Individuals with pGDM presented higher fasting glycemia (FG) (83.2 ± 13.0 vs 71.6 ± 9.4 mg/dl; p = 0.002) than others. Parity was correlated with levels of FG (r = 0.45; confidence interval: 0.03-0.75; p = 0.04). HOMA IR was higher in pGDM than in controls (p = 0.03), especially in the subgroup with normal BMI (p = 0.008) and AC < 88 cm (p = 0.009), as seen in Table 3. There was a positive correlation between AC and both HOMA IR and FG (R = 0.54; IC: 0.29-0.72; p = 0.0001). No correlations were found between HOMA IR and parity (p = 0.43), weight gain during pregnancy (p = 0.94) or weight variation after labor (p = 0.75).

**Table 3:** HOMA measurements

***VARIABLE Mean/median (SD)***	***Previous GDM (Group 1)***	***Controls (Group 2)***	***P value***
HOMA IR			
Total	2.15/1.71 (2.22)	1.23/1.04 (0.82)	**0.03**
BMI < 25	1.59/1.73 (0.54)	0.72/0.77 (0.35)	**0.008**
BMI ≥ 25	2.33/1.69 (2.54)	1.64/1.51 (0.87)	0.81
AC < 88 cm	1.59/1.73 (0.54)	0.77/0.77 (0.39)	**0.009**
AC ≥ 88 cm	2.27/1.67 (2.55)	1.22/1.06 (0.75)	0.08
LogHOMAβ			
Total	2.81/2.32 (0.44)	2.23/2.16 (0.44)	0.72
BMI < 25	2.08/1.82 (0.45)	2.02/1.93 (0.44)	0.91
BMI ≥ 25	2.34/2.38 (0.43)	2.39/2.32 (0.58)	0.77
N	25	20	

SD-Standard deviation; BMI-body mass index AC- abdominal circumference.

### Videocapillaroscopy

VC could not be done in 5 subjects (4 pGDM and 1 from control group) due to technical limitations of the method. In basal videocapillaroscopy, there was significant difference between groups in the presence of papillae rectification (68.7% vs. 20.8%, p = 0.003). Abnormal capillary morphology obtained in the basal VC is shown in Table 4. In the dynamic VC, no differences were identified between the groups (Table 5).

**Table 4:** Abnormal findings at basal videocapillaroscopy

**Abnormality**	**Previous GDM (Group 1) (%)**	**Controls (Group 2) (%)**	**HR (IC 95%)**	**p**
Granular flow	50	62, 5	0.60 (0.16-2.16)	0,52
Disordered distribution of loops	12, 5	0	8.44 (0.37- 188.63)	0,15
Loops with aberrant form	32	20, 9	1.72 (0.40- 7.32)	0,48
Short loops	25	4, 2	7.66 (0.76-76.49)	0,13
Tortuous transverse segment	31, 2	16, 6	2.27 (0.50- 10.25)	0,44
Enlarged transverse segment	50	29, 1	2.42 (0.65- 9.06)	0,20
Microectasy	37, 5	12, 5	4.20 (0.86- 20.34)	0,11
Papillae rectification	68, 7	20, 8	8.36 (1.97- 35.47)	**0,003**
N	24	19		

HR- Hazard ratio.

**Table 5:** Basal and dynamic videocapillaroscopy results

***Test***	***Previous GDM***	***Controls***	***P value***
ATSb (μ^2^)	382,58 (239.5–548.89)	420.9 (238–762.2)	p = 0.24
ATSm (μ^2^)	566.8 (335–824)	646,49 (339–1124.3)	p = 0.15
MAIt (seconds)	7 (2–12)	6 (2–10)	p = 0.71
MAIp (%)	48.1 (33.3–69.25)	50.91 (36–77.3)	p = 0.28
N	24	19	

## Discussion

This study intended to investigate possible CVD risk factors and microvascular abnormalities diagnosed through VC in women with pGDM and normal OGTT, when compared to a control group. Several interesting findings were seen. First, BMI did not differ between groups, but a higher AC was observed in those with pGDM than in controls. Verma et al. also reported similar BMI in women with and without pGDM from six to ten years after the delivery, despite higher BMI observed in pGDM in the fourth and fifth years after pregnancy [26]. In our study group, the lack of difference was observed in individuals tested 43.26 months, in average, after their labor. We found a correlation between AC and weight gain after but not during pregnancy. This finding may represent changes in fat distribution associated with the aging process or parity. A limitation in this study is the inability to determine if AC differences between groups would be present prior to pregnancy. Branchtein L et al. have shown a significant correlation between AC and glycemic levels in the OGTT in 1025 women evaluated between 21^th^ and 28^th^ weeks of pregnancy [27].

FG were higher in subjects with pGDM than others, which was more evident in those with normal BMI and AC < 88 cm. Similar differences in FG were observed previously [18,28]–[30]. There is evidence that higher FG levels, even within the normal range, may represent a marker of increased risk for CVD. Pallardo F et al. have reported an association between central obesity, triglycerides levels and arterial blood pressure with FG but not with glucose intolerance [31]. HOMA IR was also higher in women with pGDM than others, indicating that this condition is associated with increased insulin resistance, even in the absence of OGTT abnormalities. This finding was more striking in individuals with normal BMI, which leads us to believe that insulin resistance in this group occurs independently of the presence of overweight. No correlations were found between HOMA IR and parity, weight gain during pregnancy or weight variation after labor. This may indicate that insulin resistance precedes these environmental factors possibly associated with metabolic abnormalities. No differences between groups were found in the HOMAβ, what suggests that β cell dysfunction actually develops later in the natural history of T2DM. Abnormalities in insulin sensitivity but not secretion in pGDM women have been shown before [28,32].

No significant differences in the lipid profile were seen between groups, except for a tendency towards lower levels of HDL in those with pGDM, when compared to controls. Low levels of HDL are not only markers of insulin resistance, but may also of a lack of anti-atherogenic effects [33]. We cannot exclude that the lack of differences between groups in free fatty acid (FFA) levels may be due to the fact that only a basal measurement was performed. Indeed, Kousta E et al. did not find differences in basal FFA levels between women with pGDM and controls, but the former exhibited lower suppression of FFA than the latter after venous glucose tolerance tests [34].

A few authors have shown that pGDM individuals, despite being currently free from metabolic abnormalities, have higher values of markers of endothelial dysfunction, such as E-selectin and intercellular adhesion molecule 1 or ICAM-1 [35], but there is not a consensus. Higher levels of CRP and sialic acid, both markers of chronic low-grade inflammation, were also reported in women with pGDM [28,36,37]. In our study, similar levels of CRP were found in both groups and in all women this marker was within the normal range. No association was found between CRP and BMI, which might be due to the low variation of CRP levels in the whole sample, as the association between CRP and obesity has been shown before [38]. Kim et al. also found no differences in CRP and other inflammatory markers (ferritin and leucocyte count) among women with or without previous GDM [39].

Differently from Heittritter et al., who have shown higher levels of PAI-1 in women with pGDM, we did not see any difference between groups nor any association between this marker with BMI or AC. Although these results might be due to the small sample size, other hypothesis to explain this finding would be the multiethnic nature of our study group. Solano MP et al. detected differences in the association of PAI 1 and insulin resistance markers in women from different ethnic backgrounds. In that analysis, Afro-descendants women did not show any association between PAI-1 and visceral adiposity [40].

Fibrinogen levels did not differ between groups and 95% of the subjects had normal levels of this marker. Di Bennedetto et al., on the other hand, found higher levels of fibrinogen in individuals with pGDM than controls [36]. It is still unclear if fibrinogen is implicated in the pathogenesis of CVD or is a marker that reflects its appearance, but our data suggests that its raise is not an early marker of insulin resistance.

VC has shown a higher number of papillae rectification in the nail capillary bed in women with pGDM than others. Papillae rectification, disordered distribution and shortening of capillary loops were approximately 8 times more prevalent and presence of microectasias was 4 times more prevalent in women with pGDM. Hofstee et all recently show that all quantitative and certain qualitative parameters are highly reliable in terms of inter- and intra-observer agreement in VC [41].

The increase in capillary permeability is the first abnormality in diabetic microangiopathy. Abnormalities in the shape of the papillae, shortening of capillary loops and edema are markers of increased capillary permeability [42]. Microectasia is a later finding, associated with ischemia and endothelial cell damage in the capillary wall [43]. To our knowledge, this is the first study to identify VC abnormalities in women with pGDM. The prevalence of morphologic abnormalities in women with pGDM in our study group was slightly higher than that described by Halfoun et al. in healthy first degree relatives of patients with T2DM [16]. This emphasizes the importance of this finding as an early microvascular alteration in this population at risk for T2DM. A few authors have also shown early microvascular abnormalities in women with pGDM. Shannon et al. studied nondiabetic women with prior GDM and showed subclinical inflammation, hypoadiponectinemia, and early vascular dysfunction in this population suggesting an increased risk of developing CVD [28] . Anastasiou et al. have observed a endothelium-dependent vasodilation in the brachial artery [44], although a smaller study did not find any abnormality through the same technique and in vascular reactivity in the skin [45]. Aortic function assessed by ultrasonography and small vessel performance assessed by iontophoresis and laser Doppler flowmetry was also reported in these individuals. Hu et al. reported an endothelium-dependent and -independent vasodilatation impaired response of the microcirculation in women with pGDM group when compared to controls [46]. Banerjee et al. have shown that overweight women with disglycemia during pregnancy had normal vascular structure and stiffness, but detectable progressively impaired endothelium-dependent function at 2 years follow-up [47].

Those early vascular abnormalities might be a result of a “hyperglycemic legacy”. Alternatively, it is possible that some intrinsic defects in vascular function may be operating in insulin-resistant individuals, even before OGTT abnormalities become evident.

The absence of significant differences between groups in some biochemical parameters and endothelial function might be due to the characteristics of our study group, composed by healthy young women. Caballero et al. have shown important correlations between markers of endothelial damage and age in a study group that comprised relatives of patients with T2DM older than our subjects studied [48].

To summarize, women with pGDM, even with normal OGTT**,** have subtle yet significant differences in some CVD risk factors when compared to others, as well as microvascular abnormalities (papillae rectification). Although most of these individuals do not develop T2DM, they are exposed to clinical and laboratory risk factors to CVD at a young age. These women should be given counselling and appropriate advice on lifestyle, in order to decrease their risk for CVD and T2DM. Longer-term studies are necessary to verify if a pGDM would represent a marker of future increased cardiac morbidity and define the role of lifestyle and/or pharmacological interventions in these subjects.

## Competing interests

The authors declare that they have no conflict of interest.

## Authors' contribution

LZ contributed in the study design, study implementation, analysis and interpretation of data and major contribution to writing drafted the manuscript. MR drafted the manuscript. JPF contributed in the study implementation and in the manuscript writing. MLEP and JEPO contributed in the study implementation. VLCH contributed in the study design and in the interpretation of data. All authors read and approved the final manuscript.
